# Evaluation of Breast Galactography Using Digital Breast Tomosynthesis: A Clinical Exploratory Study

**DOI:** 10.3390/diagnostics11112060

**Published:** 2021-11-07

**Authors:** Juan Tao, Hao Liao, Yuan Liu, Qingsong Peng, Wenying Zhu, Shuyi Peng, Jie Liu, Leqing Chen, Fan Yang

**Affiliations:** 1 Department of Radiology, Union Hospital, Tongji Medical College, Huazhong University of Science and Technology, Wuhan 430022, China; taojuan@hust.edu.cn (J.T.); liaohao1202@163.com (H.L.); ly2354328@163.com (Y.L.); zhuwenying917@163.com (W.Z.); shuyipeng@hust.edu.cn (S.P.); liu_jie0823@163.com (J.L.); clqdyxa@163.com (L.C.); 2 Hubei Province Key Laboratory of Molecular Imaging, Wuhan 430022, China; 3 DXR Collaborations, GE Healthcare, Shanghai 201200, China; 13973118362@139.com

**Keywords:** digital breast tomosynthesis, galactography, pathological nipple discharge, full-field digital mammography, average glandular dose

## Abstract

Objectives: To compare the application value of digital breast tomosynthesis (DBT) and full-field digital mammography (FFDM) in breast galactography. Materials and Methods: A total of 128 patients with pathological nipple discharge (PND) were selected to undergo galactography. DBT and FFDM were performed for each patient after injecting the contrast agent; the radiation dose of DBT and FFDM was calculated, and the image quality was evaluated in consensus by two senior breast radiologists. Histopathologic data were found in 49 of the 128 patients. Sensitivity, specificity, positive predictive value (PPV), and negative predictive value (NPV) for both FFDM- and DBT-galactography were calculated using histopathologic results as a reference standard. Data were presented as percentages along with their 95% confidence intervals (CI). Results: The average age of the 128 patients was 46.53 years. The average glandular dose (AGD) of DBT-galactography was slightly higher than that of FFDM-galactography (*p* < 0.001). DBT-galactography was 30.7% higher than FFDM-galactography in CC view, while DBT-galactography increased by 21.7% compared with FFDM-galactography in ML view. Regarding catheter anatomic distortion, structure detail, and overall image quality groups, DBT scores were higher than FFDM scores, and the differences were significant for all measures (*p* < 0.05). In 49 patients with pathological nipple discharge, we found that the DBT-galactography had higher sensitivity, specificity, PPV, and NPV (93.3%, 75%, 97.7%, and 50%, respectively) than FFDM-galactography (91.1%, 50%, 95.3%, and 33.3%, respectively). Conclusions: Compared to FFDM-galactography, within the acceptable radiation dose range, DBT-galactography increases the sensitivity and specificity of lesion detection by improving the image quality, providing more confidence for the diagnosis of clinical ductal lesions.

## 1. Introduction

Nipple discharge (ND) only accounts for 5% of all breast symptoms in clinical practice, but it causes both anxiety and discomfort to many women [1]. Pathological nipple discharge (PND) is any unilateral, bloody or serous exudate that spontaneously appears from a single orifice [2]. The most common causes of pathologic nipple discharge are intraductal papilloma or benign ductal ectasia, and the main cause of pathologic nipple discharge involving malignant lesions is ductal carcinoma in situ (DCIS) [3]. According to the literature, the risk of malignancy increases with age [3].

Diagnostic mammography is the standard initial step in the evaluation of a patient with pathologic nipple discharge [4,5], and it has the ability to detect very small lesions in the specific duct with pathologic nipple discharge [6,7,8]. Galactography is an X-ray examination that uses mammography and an iodinated contrast to obtain imaging of the inside of the duct to evaluate lesions causing nipple discharge. Recent studies have shown that galactography helps in precisely locating masses within breast tissue and provides useful information for the surgical approach and planning [9].

Conventional galactography is a scanning two-dimensional (2D) full-field digital mammography (FFDM) [7,10,11]. It may be affected by the lesion-masking effect of overlapping normal tissue and produce false-negatives or false-positive findings. In recent years, digital breast tomosynthesis (DBT) has been widely used in breast diagnosis [12,13]. Tomosynthesis requires projections of a stationary compressed breast from multiple angles as the X-ray gantry rotates [14]. DBT enables the creation and viewing of thin-section reconstructed images to unmask obscured lesions and increased anatomic conspicuity in images of overlapping normal tissue, which may allow it to overcome the limitations of conventional 2D digital mammography in breast imaging [15]. Currently, few published articles discuss the diagnostic accuracy of DBT compared to digital mammography in nipple discharge [4,16,17]. In recent studies, Jong Yoon Lee et al. [17] applied DBT in galactography and retrospectively analyzed the application of DBT and SM 2D in catheterization in 35 cases. Marco Moschetta et al. [18] compared the diagnostic performance of DBT-galactography with that of FFD-galactography [18].

In addition, breast glandular tissue is regarded as a radiosensitive organ, and the average glandular dose (AGD) to the breast is considered to be the most important quantity to estimate the risk of radiation-induced carcinogenesis from mammography [19,20,21]. At present, there has been too little research on radiation dosages.

The primary objective of our study was to investigate the application value of DBT and FFDM in breast galactography by comparing the AGD, image quality, and diagnostic efficiency of the lesions between DBT and FFDM.

## 2. Materials and Methods

### 2.1. Patients

This study was performed in accordance with the Declaration of Helsinki and approved by the institutional ethics committee. As it was a prospective study, all participants provided written informed consent before the examinations.

**Figure 1:**
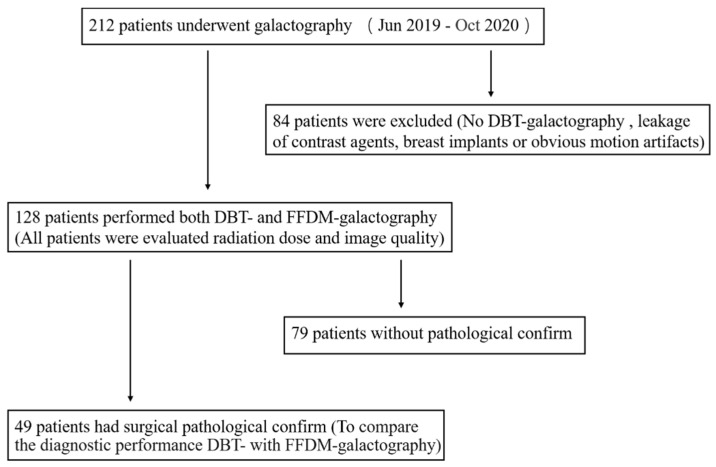
Flowcharts for studies of digital breast tomosynthesis (DBT)- and full-field digital mammography (FFDM)-galactography.

From June 2019 to October 2020, a total of 212 patients underwent galactography in our institution. Patients who disagreed with DBT were excluded, and patients with significant leakage of contrast agents in breast ducts, breast implants, or obvious motion artifacts were also excluded from this study. In the end, 128 patients with successful galactography were included in the study (Figure 1).

### 2.2. Galactography

#### 2.2.1. Galactography Operation

The galactography physician in this study had more than 6 years of experience. Galactography was performed by cannulating the secreting duct with a blunt dedicated needle (which is polished, and the edge is polished smooth) connected to a 1 mL syringe. Nonionic iodinated contrast agent was injected into the overflow catheter (our department uses 350 mg/mL iohexol), with the dose being determined by the patient’s feedback on breast tenderness and nipple contrast reflux. The usual injection dose is 0.5–1 mL with a maximum dose of 2 mL. If the contrast concentration was deemed unsatisfactory in mammography, an additional contrast injection was administered in the original discharge orifice. All participants underwent FFDM and DBT sequentially with the same breast compression. Craniocaudal and mediolateral mammograms were obtained after contrast medium injection.

#### 2.2.2. Contrast Agent

In the pre-experiment process, it was found that when a concentration of 350 mg/mL iohexol was used for ductal angiography, the visual information of the tube wall and fine bifurcation was lost due to excessively high ductal development density. We took several different concentrations of contrast agent (iohexol) for in vitro photography and found that a better image contrast could be obtained by dilating 350 mg/mL iohexol by 50%. 

### 2.3. Imaging

DBT- and FFDM-galactography were performed with a commercial system (Senographe Essential, GE Healthcare, WuHan, China). The tube was rotated using the step & shoot method. The rotation angle was ±12.5° while the breast was compressed, and the pixel size was 100 μm. The mean imaging time was less than 10 s. The projections were combined to create a full 3D tomosynthesis image set of the breast.

Two sets of mammograms (FFDM-galactography and DBT-galactography images) of the nipple discharge side were acquired, including craniocaudal (CC) and mediolateral (ML) views of both breasts. The imaging system automatically estimated the average glandular dose (AGD).

### 2.4. Imaging Analysis

All images were viewed on a dedicated mammary machine (MammoWorkstation 4.7.0, GE). All galactograms were evaluated by two radiologists with 7–8 years of experience in mammography (reader 1 and reader 2), using a 5-point Likert-type scale with respect to the following categories [22]:

- Catheter anatomic distortion (1 = very strong, 2 = strong, 3 = medium, 4 = small, 5 = negligible)
- Catheter structure detail (1 = not diagnostic, 2 = poor, 3 = moderate, 4 = good, 5 = excellent)
- Overall image quality (1 = not diagnostic, 2 = poor, 3 = moderate, 4 = good, 5 = excellent).

As overall image quality is the most important part of image analysis, the consistency between reader 1 and reader 2 was calculated. In cases of disagreement with image analysis, a senior radiologist with more than 20 years of professional breast experience was involved to define consensus, and the final score was used to compare the image quality of FFDM- and DBT-galactography.

In the comparison of imaging results, when the two readers’ evaluations of the imaging results were inconsistent, another reader with more than 20 years of professional breast experience re-interpreted the images and established consensus as the final evaluation, while being blinded to histopathology results. On both DBT and FFDM-galactography, all examinations showing filling defects, filling stops, or ductal distortion were classified as positive.

### 2.5. Statistical Analysis

All statistical analyses were performed using SPSS ver. 21.0 (SPSS Inc., Chicago, IL, USA). Categorical variables were presented as numbers (%), and continuous variables were presented as medians (Q1, Q3). The paired t-test was used for the AGD evaluation of DBT- and FFDM-galactography images. The scores on the Likert-type scale of image quality were compared between DBT- and FFDM-galactography images using the Wilcoxon rank-sum test. A value of *p* < 0.05 was considered to indicate a significant difference. The receiver operating characteristic (ROC) curve analysis was performed to illustrate the diagnostic performance of the AUC value.

## 3. Results

### 3.1. Patient Information

A total of 128 galactography images were obtained from 128 female patients, with an average age of 46.53 years (range, 27–81 years). There were 65 cases of nipple discharge on the left side and 63 cases on the right side. The minimum time of spontaneous nipple discharge was 1 day, and the maximum time was 1 year. Among all cases, yellow catheter extravasation was the most common (Table 1).

### 3.2. Average Glandular Dose (AGD)

The parameters related to AGD are shown in Table 2. In each patient’s breast, the compressed breast thickness of FFDM and DBT at CC position was similar, as was the compressed breast thickness at ML position. The calculated “mean ± SD” AGD values in CC views were 1.32 ± 0.34 mGy (range, 0.44–2.35 mGy) and 1.69 ± 0.55 mGy (range, 0.619–2.63 mGy), while the mean AGD in ML views for FFDM-galactography and DBT-galactography were 1.32 ± 0.38 mGy (range, 0.46–2.77 mGy) and 1.54 ± 0.44 mGy (range, 1.57–2.78 mGy), respectively. The AGD values of FFDM and DBT were statistically different. The radiation dose of DBT at CC and ML views increased by 30.7% and 21.7% respectively compared with FFDM.

### 3.3. Visual Image Analysis

The consistency between reader 1 and reader 2 regarding overall image quality was calculated (Table 3). We found a good agreement between the two readers.

**Figure 2:**
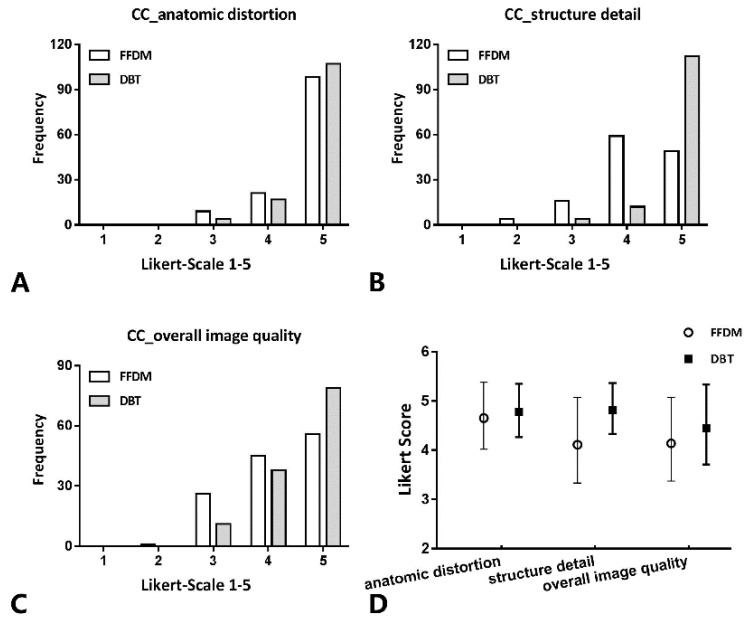
In CC views, histogram of the evaluation of catheter anatomic distortion (**A**), catheter structure detail (**B**), and overall image quality (**C**) using Likert scoring (catheter anatomic distortion (1 = very strong, 2 = strong, 3 = medium, 4 = small, 5 = negligible), catheter structure detail and overall image quality (1 = not diagnostic, 2 = poor, 3 = moderate, 4 = good, 5 = excellent); and (**D**) mean values and standard deviation of quality scores. Comparison of the image quality between DBT- and FFDM-galactography.

**Figure 3:**
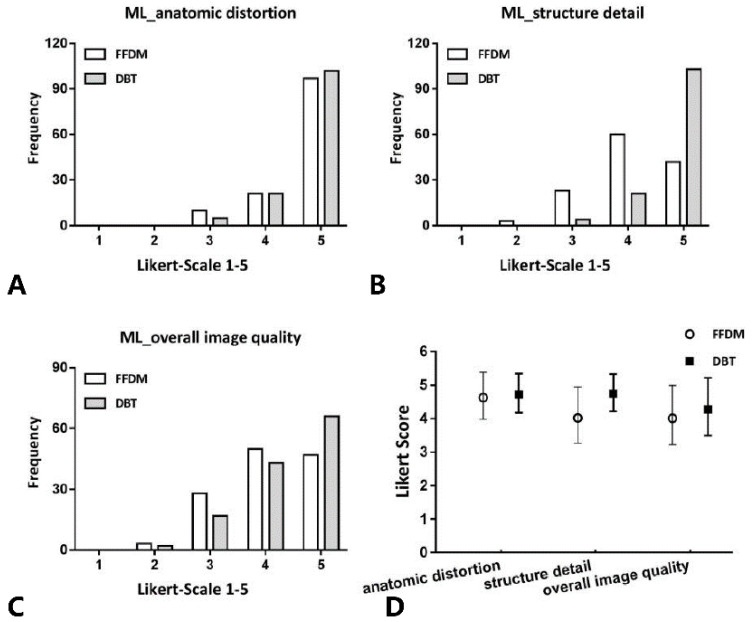
In ML views, histogram of the evaluation of catheter anatomic distortion (**A**), catheter structure detail (**B**), and overall image quality (**C**) using Likert scoring; and (**D**) mean values and standard deviation of quality scores. Comparison of the image quality between DBT- and FFDM-galactography.

**Figure 4:**
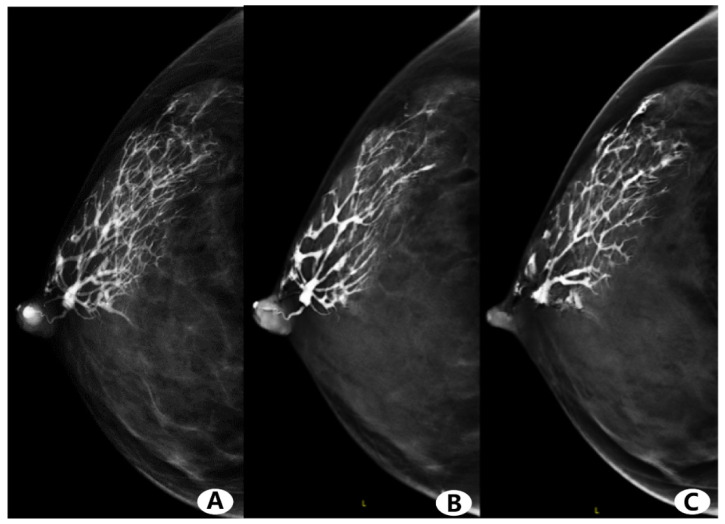
Female, 44 years old, right nipple discharge for two months. (**A**) Full-field digital mammography (FFDM)-galactography. (**B**,**C**) Digital breast tomosynthesis (DBT)-galactography. FFDM and DBT-galactography can show the distribution of the duct very well. FFDM-galactography images show obvious overlap of catheter branches, and partial branches are obscured. DBT-galactography shows more clearly the small branches than FFDM-galactography.

No severe contrast medium leaks or artifacts occurred in any participants. Figure 2 and Figure 3 show the evaluation of the image quality with respect to the catheter anatomic distortion, detail structure, and overall image quality for the CC and ML views (Figure 2A–C and Figure 3A–C, respectively). Figure 2D and Figure 3D show the mean image quality scores and their standard deviation. Regarding the catheter anatomic distortion, detail structure, and overall image quality groups, DBT scores were higher than FFDM scores, and the differences were significant for all measures (*p* < 0.05). DBT-galactography showed branches of the duct better than FFDM-galactography (Figure 4). 

### 3.4. Imaging Finding

Among the 128 patients with nipple discharge, 79 cases had no histopathological data, 61 of them received followed up visits with negative result of galactography and other imaging examinations, and 18 of the patients received follow up visits with positive results, received surgical treatment, and also were followed up for observation. After more than 1 year of follow-up, 42 out of the 61 negative patients were followed up, and the ultrasound was negative; 14 out of 18 positive patients still showed lesions in the catheter but no changes. Finally, 49 cases underwent duct excision for nipple discharge and had histopathological results.

**Figure 5:**
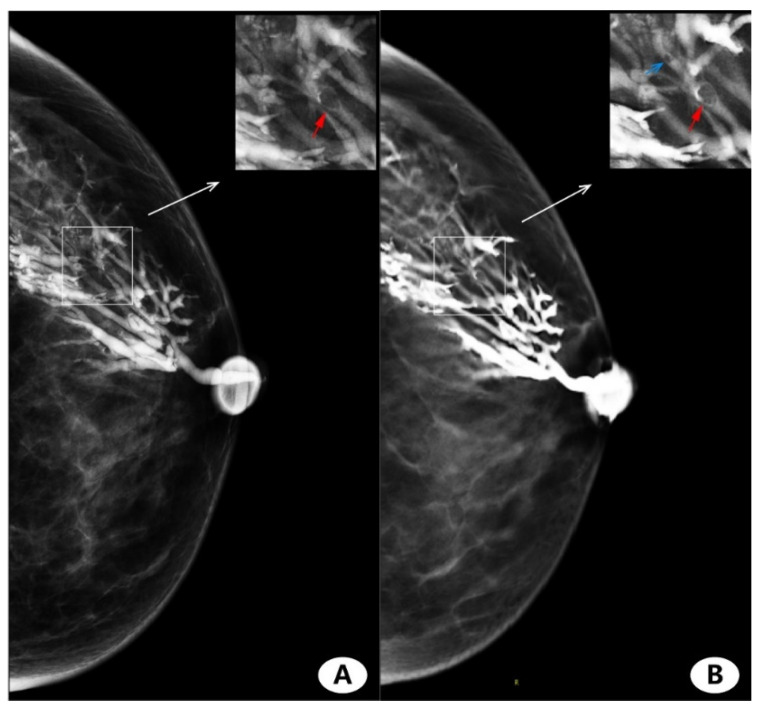
Female, 45 years, left nipple discharge for over a month. (**A**) Full-field digital mammography (FFDM)-galactography and enlarged view of lesion area. (**B**) Digital breast tomosynthesis (DBT)-galactography and enlarged view of lesion area. Both FFDM-galactography and DBT-galactography can show filling defects (red arrows) in large branches. DBT-galactography can show filling defects in small branches (blue arrow), while FFDM-galactography images cannot.

The assessment criteria for the FFDM- and DBT-galactography were normal findings, duct ectasia, ductal stenosis, single filling effect, multiple filling defects, and interrupt. DBT-galactography found more filling defects than FFDM-galactography (Figure 5). Pathologic findings were positive in 45/49 cases and negative in the remaining 4. The positive findings consisted of 26/45 (57.8%) single intraductal papilloma,14/45 (31.1%) intraductal carcinoma in situ (DCIS), and 5/45 (11.1%) invasive carcinoma (Table 1). In one false-positive patient, both FFDM- and DBT-galactography presented multiple filling defects.

FFDM-galactography and DBT-galactography were both negative in 6/49 patients while intraductal lesions were detected in 43/49 patients. Among the FFDM positive cases, two cases were pathologically negative, while only one was pathologically negative in DBT. Sensitivity, specificity, positive predictive value (PPV), and negative predictive value (NPV) for both FFDM-galactography and DBT-galactography were calculated using histological examinations of surgical specimens as a reference standard (Table 4).

## 4. Discussion

There is currently sparse literature on galactography comparing tomosynthesis with FFDM, and the study of AGD values in breast tomosynthesis as used in galactography is even rarer. Breast tomosynthesis could be used for galactography (breast ductal synthesis), which creates digital three-dimensional images to better detect intraductal lesions [16]. DBT decreases the impact of overlapping fibroglandular tissue, and DBT could yield higher diagnostic accuracy when used in combination with synthetic digital 2D full-field mammography image reconstructed from the DBT image dataset [23,24]. 

The application of X-rays has not only brought great benefits to humankind but also brought certain harm and negative effects to the health of the population [25]. The breast glandular tissue is regarded as a radiosensitive organ, and the AGD to the breast is considered to be the most important quantity to estimate the risk of radiation-induced carcinogenesis from mammography [21]. Increasingly more attention has been paid to the radiation dose [26,27,28,29]. In our study, DBT-galactography had a slightly higher radiation dose than FFDM-galactography in CC and ML views, but the increase was less than 34% of that reported in the literature [19]. In this study, the AGD maximum for FFDM-galactography and DBT-galactography imaging mode was within the range of reported literature results for digital systems [19,26,30]. In North America, FDA standards are outlined in the Mammography Quality Standard Act (MQSA), which sets a breast dose restriction of 3 mGy per acquisition of the American College of Radiology (ACR) phantom [31]. The mean calculated AGDs for both 2D and 3D imaging modes in our study were lower than these recommended limits.

In the analysis of all patients with nipple discharge, it was found that the situation of left and right effusion was roughly similar, and yellow overflow was the most common, followed by bloody overflow. DBT-galactography detected more filling defects than FFDM-galactography, and DBT-galactography was able to find the branching details of multiple ducts. 

In this study, regarding the visual effects assessment, the scores of reader 1 and reader 2 were very consistent. The consensus assessment was conducted by physicians with high seniority in order to improve the scoring system, make image scoring results more accurate and objective, and reducing human bias. FFDM- and DBT-galactography scores on the display of the catheter anatomic distortion were similar. However, DBT-galactography was better than FFDM-galactography at displaying the structure detail and overall image quality, especially at displaying the branching structure of the catheter.

In our study, one case was found to have lesions in both DBT-galactography and histopathology, while FFDM-galactography was shown to be negative. In another case, both DBT-galactography and histopathology were negative, while FFDM showed an intraductal secondary branch filling defect. There was another case that DBT and FFDM showed multiple fill defects with a pathology negative and positive in the catheter may be caused by secretions. In 49 patients with pathological nipple discharge, surgical pathological confirmation found that the DBT-galactography had higher sensitivity, specificity, PPV, and NPV than FFDM-galactography. This is consistent with the findings of Moschetta et al. [18] that DBT-galactography is more sensitive, and that the application of DBT-galactography in catheter increases the confidence of diagnosis. 

Our study had several limitations. First, all of our data came from a single center, and FFDM and DBT images were generated by using a commercially available software module from a single vendor (Senographe Essential, GE); thus, the generalizability of the results to images obtained using other vendors’ reconstruction algorithms and other types of practices would have to be tested. In addition, another limitation was our small patient sample. Furthermore, ductal secretions may interfere with the correct diagnosis of the lesion.

## 5. Conclusions

DBT-galactography has a slightly higher radiation dose than FFDM-galactography and significantly lower than international standards. Considering its benefits, this radiation dose is acceptable. Compared with FFDM-galactography, DBT-galactography could improve the diagnostic efficacy without significantly increasing the radiation dose. This new procedure can better show lesions, especially in multiple ducts. Further studies are needed to validate and generalize the findings of galactography in combination with DBT for both diagnostic and screening populations.

## Figures and Tables

**Table 1:** Characteristics of patients.

Characteristics	*n* = 128	*n* = 49
**Age (years)**	46.53 ± 9.4 (27–81)	48.78 ± 9.5 (28–71)
**Gender**	female	female
**Discharge side**		
Left side	65	23
Right side	63	26
**Discharge color**		
Yellowish	66 (51.6%)	26 (53.1%)
Bloody	39 (30.5%)	22 (44.9%)
Clear	15 (11.7%)	1 (2.0%)
White	8 (6.2%)	0 (0%)
**Pathologic findings (*n* = 49)**		
Negative		4
Intraductal papilloma		26
Intraductal carcinoma in situ		14
Invasive carcinoma		5

**Table 2:** Comparison of AGD and related parameters between FFDM-galactography and DBT-galactography.

Projection		kVp	mAs	Times (ms)	CBT (mm)	Compression Force (daN)	AGD (mGy)
CC	FFDM	28.9 ± 1.16	64.19 ± 16.60	937.57 ± 321.80	48.78 ± 11.38	6.22 ± 2.95	1.32 ± 0.34
	DBT	29.12 ± 1.03	65.09 ± 20.15	987.03 ± 362.14	48.73 ± 11.22	6.34 ± 3.43	1.69 ± 0.55
	T values	−1.982	−0.613	−2.289	0.602	−0.731	−9.590
	*p* values	0.050 *	0.541	0.024 ^*^	0.548	0.466	<0.001 **
ML	FFDM	28.61 ± 1.14	63.59 ± 16.92	879.60 ± 304.26	45.08 ± 9.94	6.46 ± 3.77	1.32 ± 0.38
	DBT	28.91 ± 1.08	59.43 ± 15.61	874.80 ± 290.39	44.99 ± 9.94	6.37 ± 3.80	1.54 ± 0.44
	T values	−3.59	2.082	0.225	0.973	0.712	−6.161
	*p* values	0.001 *	0.039 *	0.822	0.332	0.478	<0.001 *

* *p* < 0.05; ** *p* < 0.001; *t*-test, two-tailed. *n* = 128, craniocaudal (CC), mediolateral (ML), compressed breast thickness (CBT), compression force, and average glandular dose. AGD per exposure for each projection in 2D imaging and DBT mode. Data are presented as mean ± SD.

**Table 3:** Interobserver agreement for overall image quality.

		Reader 1	Reader 2	Kappa Value	*p* Value
CC	FFDM	4.220 ± 0.793	4.200 ± 0.787	0.855	<0.001
	DBT	4.530 ± 0.651	4.590 ± 0.633	0.814	<0.001
ML	FFDM	4.100 ± 0.821	4.100 ± 0.831	0.754	<0.001
	DBT	4.370 ± 0.730	4.450 ± 0.719	0.813	<0.001

Note: Data are presented as means ± SD. Interobserver agreement is presented as Kappa value.

**Table 4:** Diagnostic performance of digital breast tomosynthesis (DBT)-galactography and full-field digital mammography (FFDM)-galactography in 49 patients with pathologic nipple discharge.

	Sensitivity	Specificity	PPV	NPV	Efficiency	χ^2^	*p*	ROC Area
FFDM	41/45 (91.1%)	2/4 (50%)	41/43 (95.3%)	2/6 (33.3%)	43/49 (87.8%)	5.778	0.108	0.706
DBT	42/45 (93.3%)	3/4 (75%)	42/43 (97.7%)	3/6 (50%)	45/49 (91.8%)	15.963	0.001 *	0.842

* *p* < 0.05. *n* = 49, positive predictive value (PPV), negative predictive value (NPV).

## Data Availability

The data that support the findings of this study are available from the corresponding author upon reasonable request.
